# Characterization, Classification and Authentication of Spanish Blossom and Honeydew Honeys by Non-Targeted HPLC-UV and Off-Line SPE HPLC-UV Polyphenolic Fingerprinting Strategies

**DOI:** 10.3390/foods11152345

**Published:** 2022-08-05

**Authors:** Víctor García-Seval, Clàudia Martínez-Alfaro, Javier Saurina, Oscar Núñez, Sònia Sentellas

**Affiliations:** 1 Department of Chemical Engineering and Analytical Chemistry, Universitat de Barcelona, Martí i Franquès 1-11, E-08028 Barcelona, Spain; 2 Research Institute in Food Nutrition and Food Safety, Universitat de Barcelona, Recinte Torribera, Av. Prat de la Riba 171, Edifici de Recerca (Gaudí), Santa Coloma de Gramenet, E-08921 Barcelona, Spain; 3 Serra Húnter Fellow, Generalitat de Catalunya, Rambla de Catalunya 19-21, E-08007 Barcelona, Spain

**Keywords:** blossom honeys, honeydew honeys, HPLC-UV, fingerprinting, chemometrics

## Abstract

Honey is a highly consumed natural product produced by bees which is susceptible to fraudulent practices, some of them regarding its botanical origin. Two HPLC-UV non-targeted fingerprinting approaches were evaluated in this work to address honey characterization, classification, and authentication based on honey botanical variety. The first method used no sample treatment and a universal reversed-phase chromatographic separation. On the contrary, the second method was based on an off-line SPE preconcentration method, optimized for the isolation and extraction of polyphenolic compounds, and a reversed-phase chromatographic separation optimized for polyphenols as well. For the off-line SPE method, the use of HLB (3 mL, 60 mg) cartridges, and 6 mL of methanol as eluent, allowed to achieve acceptable recoveries for the selected polyphenols. The obtained HPLC-UV fingerprints were subjected to an exploratory principal component analysis (PCA) and a classificatory partial least squares-discriminant analysis (PLS-DA) to evaluate their viability as sample chemical descriptors for authentication purposes. Both HPLC-UV fingerprints resulted to be appropriate to discriminate between blossom honeys and honeydew honeys. However, a superior performance was accomplished with off-line SPE HPLC-UV polyphenolic fingerprints, being able to differentiate among the different blossom honey samples under the study (orange/lemon blossom, rosemary, thyme, eucalyptus, and heather). In general, this work demonstrated the feasibility of HPLC-UV fingerprints, especially those obtained after off-line SPE polyphenolic isolation and extraction, to be employed as honey chemical descriptors to address the characterization and classification of honey samples according to their botanical origin.

## 1. Introduction

Honey is a highly consumed natural product produced by bees (*Apis mellifera*), widely appreciated not only for its taste and nutritional value [1] but also for its health benefits [2]. It is mainly produced from carbohydrate-rich exudates generated by plants, although it also contains other components provided by the honeybees or generated by means of biochemical reactions within the honey maturation process in the hives. Despite the great variability of honeys, they can be classified into two main groups according to the source of the carbohydrate-rich exudates employed by the honeybees: blossom and honeydew honeys [3,4]. Blossom honey is produced from the nectar of flowers, while honeydew honey is generated from secretions of living parts of the plants or excretions produced by plant-sucking insects [5]. These two types of honey have very different properties and characteristics [2,3,4,6]. For example, honeydew honeys are richer in several bioactive substances such as phenolic compounds, some proteins, and amino acids in comparison to blossom honeys, contributing to their higher antioxidant and antimicrobial properties [2]. Honeys can also be classified depending on their botanical origin and only if the whole product, or most of it, comes from the indicated botanical source and keeps the microscopic, physicochemical, and organoleptic properties of that source [5]. Accordingly, monofloral honeys are normally defined as those where more than 45% of the pollen comes from a specific plant species and can then be named by its botanical variety. However, this percentage may change in some cases, for example, to higher than 90% in the case of chestnut honey, or down to 10–20% for citrus, lavender, thymus, and rosemary honeys, among others [7]. In contrast, honey is considered polyfloral or multifloral when a variable percentage of pollen coming from different plants is used for its production. Obviously, honey characteristics will also depend on many other factors such as the geographical region of production, giving place to a widely variable food product.

Honey is among the food products most likely to be adulterated, for example by the addition of syrups and other sugar-based adulterants, to obtain an economic benefit [8,9,10]. In addition, the assessment of honeys’ botanical variety as well as their geographical origin has become also an important authenticity issue for society because of the great differences in the honey characteristics and, thus, the price that honey can reach. For example, honey produced in China or South American countries is typically cheaper than honey produced in some European countries, mainly due to its lower quality and the recently reported fraud incidents. Moreover, honey produced in Southern European countries are in greater demand because of the unique and recognized characteristics attributed to the Mediterranean climate. Correct attribution of the main botanical origin is also an important authenticity issue because of the great differences in health benefits depending on the honey composition. For all these reasons, the development of simple, fast, and reliable analytical methodologies for the characterization, classification, and authentication of honey is needed [11].

Nowadays, two main analytical approaches are employed when addressing food authenticity issues: targeted and non-targeted strategies. Targeted methodologies focus on the determination of known selected chemicals (or specific parameters) whose content (or value) in the studied matrix is typically well known. In general, these compounds belong to the same family, being structurally similar. Since the whole analysis relies on them, the full analytical method (i.e., sample treatment, isolation and extraction, analytical determination, acquisition conditions, etc.) is developed and optimized to obtain satisfactory performance. Bioactive chemical compounds typically found in honey samples can then be employed as targeted markers to assess honey authenticity [12,13], such as phenolic and polyphenolic compounds [4,14,15,16,17,18,19,20], sugars [4,21], volatile compounds [22], etc. However, the main drawback of targeted approaches is the requirement of chemical standards for quantitation purposes (and to guarantee compound identity), which is also a difficult and time-consuming task when dealing with complex sample matrices such as in the case of honey. For this reason, recently, non-targeted (fingerprinting) methodologies are gaining relevance to address food authenticity issues. These methods focus on registering as many instrumental responses as possible from the analyzed samples without the requirement of having information about the known or unknown compounds responsible for the obtained responses. Then, the registered fingerprint is employed as the source of sample chemical descriptors to address classification and authentication by means of chemometric methodologies. Simple sample treatment procedures are typically employed with these techniques aiming at not modifying the obtained chemical information from the sample during its treatment. Nuclear magnetic resonance (NMR) [23,24], near-infrared (NIR) [25,26,27,28], fluorescence [29], and ultraviolet (UV) [28,30,31,32,33] spectroscopies, among others, have been widely employed as fingerprinting methodologies to address honey authenticity issues and to prevent and detect frauds. Chromatographic fingerprinting using both liquid chromatography and gas chromatography (for the analysis of non-volatile and volatile compounds, respectively), often coupled with mass spectrometry (MS), has also been described for the characterization and authentication of honey [34,35,36].

In this work, two HPLC-UV fingerprinting approaches were developed for the characterization, classification, and authentication of honey samples. The first one, established as a non-targeted HPLC-UV chromatographic fingerprint, was based on a honey dilution as the unique sample treatment and a chromatographic separation under universal gradient elution conditions. In the second one, we aimed to develop a specific sample treatment based on solid-phase extraction (SPE) using HLB cartridges for the isolation, extraction, and preconcentration of polyphenols in honey, and the obtained extracts were then analyzed by an HPLC-UV method optimized for the separation of polyphenols. The instrumental response obtained by this off-line SPE HPLC-UV method was used as the data for this polyphenolic fingerprinting strategy. Both methodologies were applied to the analysis of 136 honey samples of different botanical origins, including blossom honeys (orange/lemon blossom, rosemary, thyme, eucalyptus, heather, and multifloral honey) and honeydew honeys (mountain, forest, and holm oak), obtained from different Spanish geographical regions. The obtained HPLC-UV fingerprints were then used as honey chemical descriptors and subjected to a non-supervised principal component analysis (PCA) and a supervised partial least squares-discriminant analysis (PLS-DA) to address honey classification and authentication.

## 2. Materials and Methods

### 2.1. Reagents and Chemicals

Methanol (Chromosolv™ for HPLC, ≥99.9%) and acetonitrile (UHPLC supergradient ACS) were obtained from PanReac AppliChem (Barcelona, Spain), formic acid (≥98%) from Sigma-Aldrich (St Louis, MO, USA), and hydrochloric acid (37%) from Fisher Chemical (Geel, Belgium). Water was purified with an Elix 3 coupled to a Milli-Q system from Millipore Corporation (Millipore, Bedford, MA, USA), and was filtered through a 0.22 µm nylon membrane integrated into the Milli-Q system.

All the polyphenolic standards used in this work were of analytical grade. Gallic acid, *p*-hydroxybenzoic acid, vanillic acid, caffeic acid, *p*-coumaric acid, hesperidin, luteolin, and apigenin were obtained from Sigma-Aldrich, and ferulic acid and kaempferol from Fluka (Steinheim, Germany).

### 2.2. Honey Samples

A total of 136 different blossom and honeydew honeys from several botanical origins (varieties) and different Spanish geographical regions of production were purchased in supermarkets and local markets in Spain. Two heather honeys were directly provided by Miel de Braña (León, Spain). The studied blossom honey varieties included orange/lemon blossom (BL), eucalyptus (EU), rosemary (RO), thyme (TH), and heather (HE) samples, while holm oak (HO), mountain (MO), and forest (FO) were the honeydew honey varieties analyzed. Multifloral (MF) honeys were also considered. Samples of each variety were obtained from different Spanish geographical origins, namely Andalusia (AN), Aragon (AR), Asturias (AS), Basque Country (BC), Cantabria (CN), Castile La Mancha (CM), Castile and Leon (CL), Catalonia (CT), Extremadura (E), Balearic Islands (BI), Navarre (N), Spain (S), and Spain and others (SO) (Table 1).

### 2.3. Sample Preparation

#### 2.3.1. Honey Samples Analyzed by the Non-Targeted HPLC-UV Fingerprinting Method

Honey samples analyzed with the universal non-targeted HPLC-UV fingerprinting method were prepared as follows: approximately 1 g of honey was weighed in a 15 mL PTFE centrifuge tube (Serviquimia, Barcelona, Spain) and dissolved in 10 mL of Milli-Q water by mixing the contents using a VibraMix Vortex from OVAN (Barcelona, Spain). Then the samples were centrifuged for 5 min at 3500× *g* rpm (Rotina 420 Centrifuge from Hettich, Tuttlingen, Germany) to separate any non-soluble particles (that may include bee bread, pollen, proteins, etc.). The obtained extracts were then diluted with methanol in a 1:1 ratio, filtered through 0.45 µm syringe membrane filters (FILTER-LAB, Barcelona, Spain), and kept at 4 °C until analysis. Honey samples were randomly analyzed. In the case of crystallized honeys (which is a normal state of natural raw honeys), they were first introduced in a water bath at 45 °C until they melted. After their homogenization and cooling, they were treated following the same procedure.

50 µL of each diluted extract were mixed to prepare a quality control (QC) sample that was used to evaluate the repeatability and the robustness of the proposed non-targeted HPLC-UV fingerprinting methodology and that the chemometric results were not affected by instrumental drifts.

#### 2.3.2. Honey Samples Analyzed by the Off-Line SPE HPLC-UV Polyphenolic Fingerprinting Method

Honey samples analyzed with the off-line SPE HPLC-UV polyphenolic fingerprinting method were prepared as follows: approximately 1 g of honey was weighed in a 15 mL PTFE centrifuge tube and dissolved in 10 mL of Milli-Q water acidified with hydrochloric acid (pH 2.0), the contents mixed with a VibraMix Vortex (Labopolis, London, UK). Then, the aqueous honey solutions were extracted using an off-line SPE method developed for the recovery of polyphenols using Oasis HLB (3 mL, 60 mg) cartridges from Waters (Milford, MA, USA). Cartridges were conditioned with 3 mL of methanol and 3 mL of acidified water (HCl, pH 2.0). Then, the 10 mL of the honey aqueous solutions were loaded into the Oasis HLB cartridges at a flow rate of 1–2 mL min^−1^ using a Visiprep System (Supelco, Bellefonte, PA, USA). The cartridges were then washed with 3 mL of acidified water (HCl, pH 2.0) and dried with air. The retained bioactive compounds (among them polyphenols) were eluted with 6 mL of methanol into 15 mL PTFE centrifuge tubes, and the eluates were evaporated to dryness at 40 °C under a nitrogen stream using a Caliper TurboVap LV evaporator (Marshall Scientific, Hampton, NH, USA). Finally, the extracts were re-dissolved with 1 mL of water:acetonitrile (95:5 *v*/*v*) solution, filtered through 0.45 µm syringe membrane filters (FILTER-LAB), and kept at 4 °C until analysis.

A volume of 50 µL of each extract were mixed to prepare a quality control (QC) sample that was used to evaluate the repeatability and robustness of the proposed HPLC-UV fingerprinting method and to ensure that chemometric results are not affected by instrumental drifts.

### 2.4. Instrumentation

All experiments were performed on an Agilent 1100 Series HPLC instrument (Waldbronn, Germany) equipped with a binary pump (G1312A model), an automatic sample injector (WPALS G1367A model), a diode-array detector (G1315B model), and a PC with the Agilent Chemstation software. HPLC-UV fingerprints were obtained on a Kinetex**^®^** C18 reversed-phase column (100 × 4.6 mm i.d., 2.6 µm partially porous particle size) from Phenomenex (Torrance, CA, USA) under different gradient elution conditions given below.

#### 2.4.1. Non-Targeted HPLC-UV Fingerprinting Method

The gradient elution profile was created with 0.1% formic acid in water (solvent A) and acetonitrile (solvent B) as mobile phase components, at a flow rate of 400 µL min^−1^, as follows: 0–5 min, initial conditions at 3% solvent B; 5–13 min, linear gradient elution from 3 to 95% solvent B; 13–15 min, isocratic elution at 95% solvent B; 15–15.5 min, back to initial conditions at 3% solvent B; and 15.5–20 min, column equilibration at initial conditions. The injection volume was 5 µL and the UV acquisition was carried out at 280 nm.

#### 2.4.2. Off-Line SPE HPLC-UV Polyphenolic Fingerprinting Method

Gradient elution conditions for the off-line SPE HPLC-UV polyphenolic fingerprinting method were performed using 0.1% formic acid in water (solvent A) and acetonitrile (solvent B) as mobile phase components at a flow rate of 800 µL min**^−^**^1^, using the following elution program: 0–1 min, linear gradient elution from initial conditions (5% solvent B) to 10% solvent B; 1–4 min, linear gradient elution from 10 to 16% solvent B; 4–8 min, isocratic elution at 16% solvent B; 8–8.5 min, linear gradient elution from 16 to 25% solvent B; 8.5–13.5 min, linear gradient elution from 16 to 60% solvent B; 13.5–16 min, linear gradient elution from 60 to 100% solvent B; 16–16.5 min, isocratic elution at 100% solvent B; 16.5–16.6 min, back to initial conditions at 5% solvent B; and 16.6–22 min, column equilibration at initial conditions. The injection volume was 5 µL and the UV acquisition was carried out at 280 nm.

### 2.5. Data Analysis

All the honey extract samples were analyzed randomly with the proposed HPLC-UV method to minimize the influence of instrumental drifts on the results. A QC and an instrumental blank (Milli-Q water) were injected after each ten samples. The obtained HPLC-UV fingerprinting raw data was exported to a spreadsheet using Unichrom software from New Analytical Systems (Minsk, Belarus). Different data matrices were built from both non-targeted HPLC-UV and off-line SPE HPLC-UV polyphenolic fingerprints. Data was autoscaled to achieve the same weight for each variable by minimizing differences in the magnitude and amplitude of their scales.

Data matrices were subjected to a principal component analysis (PCA) and a partial least squares-discriminant analysis (PLS-DA) using SOLO 8.6 chemometric software from Eigenvector Research (Manson, WA, USA). Details of the theoretical background of these chemometric methods are given elsewhere [37].

The PCA was employed as an exploratory method to evaluate the distribution of the analyzed honey samples and the QC sample behavior. The PLS-DA was employed as supervised sample classificatory methods according to honey botanical and geographical production origin. The X-data matrix for the PCA was built considering the chromatograms recorded at 280 nm for each sample, i.e., the absorbance value at a specific time over the entire chromatogram was given for each sample (Figure S1 in the Supplementary Material). In the PLS-DA, the previous X-data matrix was employed (without QCs), together with a Y-data matrix defining each sample class (honey botanical origin or honey geographical production region). The validation of the PLS-DA model was carried out by using 70% of the samples (randomly selected) as the calibration set and the remaining 30% of the samples as the prediction set. The number of LVs used to build the PLS-DA models was established by the first relevant minimum of the cross-validation (CV) error from the Venetian blind approach when the matrix included more than 20 samples, and the leave-one-out approach for studies with less than 20 samples. The ellipses delimiting areas in the score plots of the PCA and the PLS-DA analyses were drawn manually to facilitate the visualization of the differentiated sample clusters.

## 3. Results and Discussion

### 3.1. Non-Targeted HPLC-UV Fingerprinting Methodology

#### 3.1.1. Chromatographic Fingerprints

**Figure 1:**
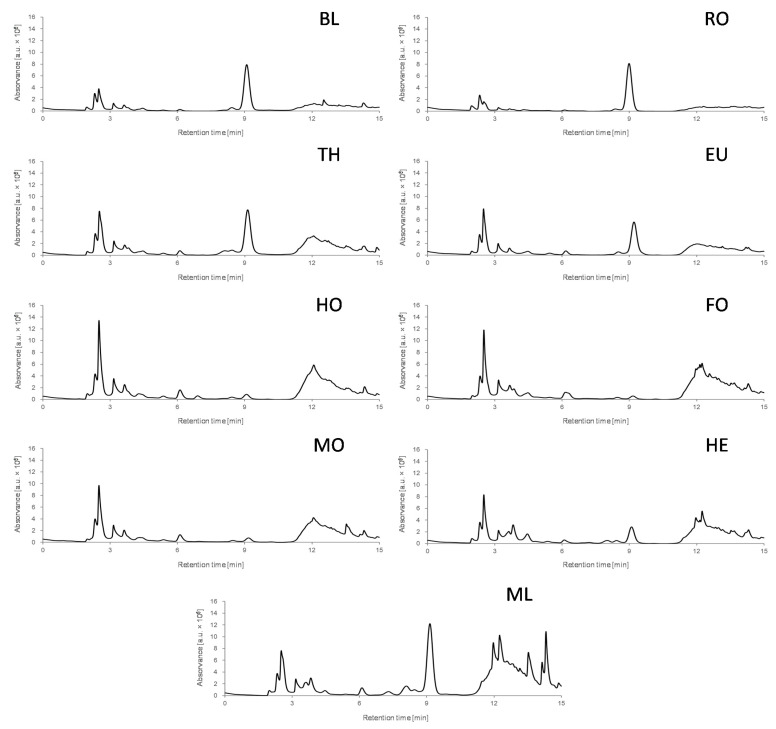
Non-targeted HPLC-UV chromatographic fingerprints at 280 nm for one selected blossom and honeydew honey sample of each botanical origin under study. BL: Orange/lemon blossom; EU: Eucalyptus; FO: Forest; HE: Heather; HO: Holm oak; MF: Multifloral; MO: Mountain; RO: Rosemary; TH: Thyme.

The main objective of this contribution was to exploit non-targeted HPLC-UV chromatographic fingerprints as potential sample chemical descriptors to address the characterization and classification of blossom and honeydew honeys according mainly to their botanical origin. These fingerprinting approaches focus on registering instrumental signals (in this case the absorbance) as a function of the chromatographic retention time, without the requirement of specific information about the components present in the samples and trying to monitor as much instrumental discriminant signals as possible. With this aim, a universal non-targeted HPLC-UV chromatographic fingerprinting methodology was first developed. For that purpose, a simple honey sample treatment was applied, consisting of dissolving 1 g of honey in 10 mL of water, and the posterior dilution with methanol in a 1:1 ratio before HPLC-UV analysis. Chromatographic fingerprints were then obtained by typical reversed-phase separation using a porous-shell C18 column and 0.1% aqueous formic acid and acetonitrile as mobile phase components. As a non-targeted HPLC-UV approach was intended, a universal gradient program, increasing acetonitrile from 3% to 95%, was employed (Section 2.4.1). A total of 136 blossom and honeydew honey samples were then analyzed with the proposed method. The non-targeted HPLC-UV chromatographic fingerprints (registered at 280 nm) for one selected honey sample of each botanical origin are depicted in Figure 1. We experimentally verified that the richest chromatograms in terms of number and intensity of peaks were recorded at 280 nm. At higher wavelengths, for example at 325 or 370 nm, at which other phenolic compounds are often monitored, the honey fingerprints were poorer. In contrast, at shorter wavelengths, the signal/noise ratio was worse and the drift of the chromatographic baseline also made the characterization more difficult, so it is not a recommended situation either.

As can be seen, although similar chromatographic fingerprints seem to be obtained, important differences in both signal profiles and intensities can be observed, especially in three chromatographic regions, i.e., sections from 1.5 to 5 min, from 6 to 10 min, and from 11 to 15 min. Three different fingerprinting patterns are distinguished after visual comparison. First, orange/lemon blossom, rosemary, thyme, and eucalyptus honey samples show similar fingerprints characterized by an important peak signal at around 9 min, and similar profiles but with different intensities in the chromatographic sections from 1.5 to 5 min, and from 11 to 15 min. Another fingerprinting pattern is the one observed with holm oak, forest, mountain, and heather honey samples, characterized in this case by richer peak signals and high intensities in the chromatographic regions from 1.5 to 5 min and from 11 to 15 min. The presence of the peak signal at around 9 min in heather samples, with a higher intensity than in holm oak, forest, and mountain honeys, must also be highlighted. However, this signal is not so intense as the one observed for the honey group constituted by orange/lemon blossom, rosemary, thyme, and eucalyptus honeys. Finally, multifloral honeys show a fingerprinting pattern richer in both peak signals and intensities in the three chromatographic regions commented, which can be attributed to the contribution of different botanical origins. Besides, the non-targeted HPLC-UV fingerprints were quite reproducible within the same honey botanical origin, which pointed out their suitability to be used as chemical descriptors to characterize and classify the analyzed honey samples.

#### 3.1.2. Chemometric Data Analysis

**Figure 2:**
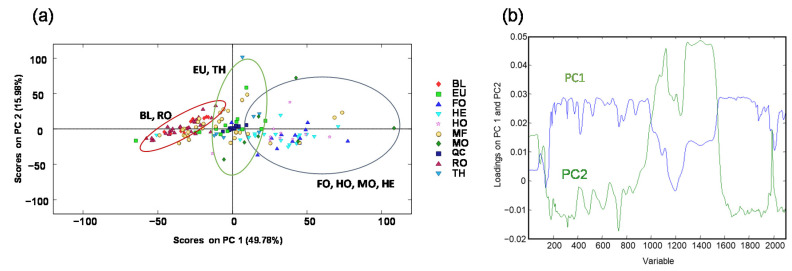
PCA score plot of PC1 vs. PC2 (**a**) and plot of loadings of PC1 and PC2 (**b**) when using non-targeted HPLC-UV chromatographic fingerprints (at 280 nm) as honey chemical descriptors. BL: Orange/lemon blossom; EU: Eucalyptus; FO: Forest; HE: Heather; HO: Holm oak; MF: Multifloral; MO: Mountain; RO: Rosemary; TH: Thyme.

Once the non-targeted HPLC-UV fingerprints of all the honey samples were obtained with the proposed method, the robustness and reproducibility of the method and the characterization and classification of the analyzed honey samples were assessed by a PCA and a PLS-DA. First, a non-supervised PCA study was performed with non-targeted HPLC-UV chromatographic fingerprints as sample chemical descriptors. Figure 2 depicts the obtained PCA results. The reproducibility of the proposed non-targeted HPLC-UV method, and the robustness of the obtained chemometric results are confirmed by the behavior of the QCs, which appeared all clustered together in the middle of the plot, showing that no instrumental drifts affected the results.

Regarding the sample distribution, they tend to be grouped according to their botanical origin, although some botanical classes are quite overlapped. However, a deep study of the plot suggests that samples tend to be more or less distributed in three different regions of the plot according to similarities in their botanical origin, being this distribution mainly explained by PC1. Orange/lemon blossom (BL) and rosemary (RO) honeys are well grouped in the left part of the plot presenting negative PC1 values. In the middle area of the plot, thyme (TH) and eucalyptus (EU) honey varieties are found, even though they are slightly more dispersed. In contrast, forest (FO), holm oak (HO), mountain (MO), and heather (HE) honey varieties are more widely distributed but located within the right area of the score plot (with positive PC1 values), clearly differentiated from BL, RO, TH, and EU samples. Finally, multifloral (MF) honey samples are spread out throughout the plot, as expected, due to their higher variability in terms of botanical origin. These results show that, in general, a clear separation between honeydew honeys (MO, HO, and FO samples), located at the right part of the plot, and blossom honeys (BL, RO, TH, and EU samples), located at the center and left part of the plot, can be accomplished by employing non-targeted HPLC-UV fingerprints as chemical descriptors. This sample differentiation could be related to the different polyphenolic content, characteristic of each group of samples. Honeydew honeys normally have more phenolic acids than blossom honeys, which by contrast are richer in flavonoids. Besides, the total phenolic content (TPC) is also higher in honeydew honeys [38]. This may justify the fact that all honeydew honeys (HO, MO, and FO) appear grouped in the PCA plots. HE honeys (classified as blossom honey) deserve special attention. Samples belonging to this variety are grouped with the honeydew honeys (right area of the plot) instead of with the blossom honeys. This fact can be explained by the similarities of this variety with honeydew honeys, such as their dark color or the TPC, which is also particularly high. In addition, the MO honeys studied in this work are mostly made from holm oak, pine, oak, heather, and chestnut. Considering that the chestnut variety is also a kind of blossom honey with the same behavior as the heather ones, it is also reasonable that MO samples are also grouped with the HO, FO, and HE varieties. Finally, the differentiation of blossom honeys in two groups (BL/RO and TH/EU) may be mainly due to differences in their flavonoid composition [39].

**Figure 3:**
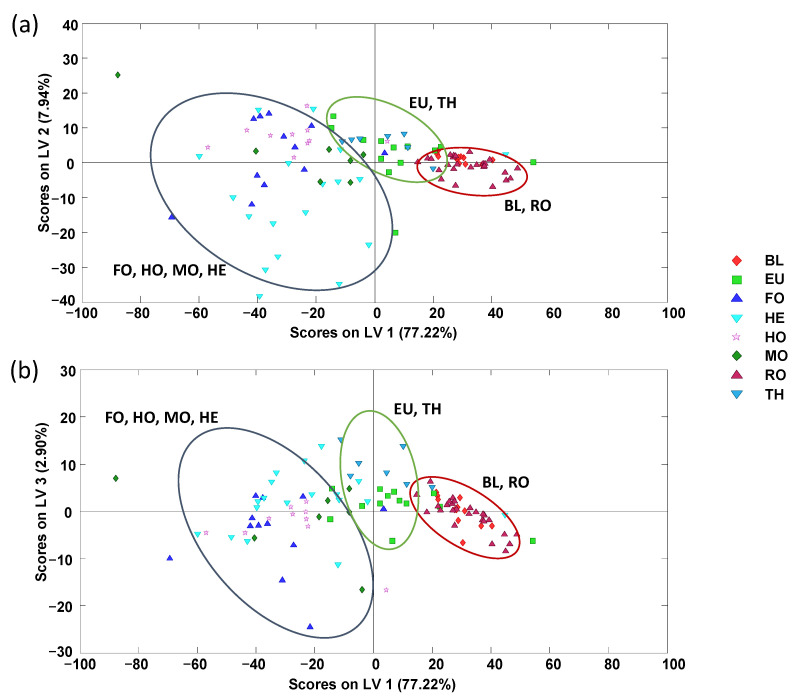
Supervised PLS-DA score plots of (**a**) LV1 vs. LV2 and (**b**) LV1 vs. LV3 when using non-targeted HPLC-UV chromatographic fingerprints (at 280 nm) as honey chemical descriptors for botanical origin classification (3 LVs were used to build the model). QCs and multifloral honeys were not considered. BL: Orange/lemon blossom; EU: Eucalyptus; FO: Forest; HE: Heather; HO: Holm oak; MO: Mountain; RO: Rosemary; TH: Thyme.

The characterization and classification of the analyzed honey samples were also evaluated by the supervised PLS-DA chemometric method using the non-targeted HPLC-UV fingerprints. To simplify the model, QCs and multifloral honey samples were not considered. The obtained PLS-DA score plots of LV1 vs. LV2 and LV1 vs. LV3 are depicted in Figure 3.

As can be seen, the PLS-DA confirms the results obtained by the PCA, showing that the honey samples tend to be grouped according to their botanical variety within three main regions through LV1, being in this case honeydew honeys distributed in the left area of the plots (with negative LV1 values), TH and EU honeys at the central area, and BL and RO honeys at the right section of the plots (with positive LV1 values). Finally, a paired PLS-DA model based on blossom honeys against honeydew honeys was validated to evaluate the classification capacity of the proposed fingerprints. For that purpose, 70% of the samples were randomly selected as the calibration set and the remaining 30% of the samples were used as the prediction set. The results are sown in Figure S2 (Supplementary Materials). As can be seen, acceptable results were obtained with sensitivity values of 0.918, 0.904, and 0.857, and specificity values of 0.870, 0.826, and 1.000, for calibration, cross-validation, and prediction, respectively. Thus, low classification errors, with values of 9% and 12% for calibration and prediction, respectively, were obtained.

To evaluate the classification capacity based on non-targeted HPLC-UV chromatographic fingerprints, PLS-DA models considering blossom or honeydew honeys, independently, were also studied. For blossom honeys, the obtained PLS-DA score plot of LV1 vs. LV2 (Figure S3 of the Supplementary Materials) shows, as expected, that heather honeys (exhibiting positive LV1 values) can clearly be discriminated from the other blossom types because of their notable differences regarding bioactive compounds, color, etc., as previously commented. Regarding the other blossom honeys, two groups can be considered. First, thyme and eucalyptus honeys, which are more dispersed and located in the right-middle area of the plot, and then orange/lemon blossom and rosemary honeys which are more grouped and located in the left area of the plot (with negative LV1 values). Regardless of the obtained honey sample distribution, a clear discrimination between the five blossom honey varieties was not accomplished. When focusing only on honeydew honeys, the obtained PLS-DA score plot of LV1 vs. LV2 (Figure S4 of the Supplementary Materials) shows that the three varieties cannot be differentiated by employing non-targeted HPLC-UV fingerprints as sample chemical descriptors, although it seems that the holm oak variety tends to be somewhat discriminated from the other two (forest and mountain varieties).

A PLS-DA model to evaluate the classification of the analyzed honey samples based on geographical origin (see Table 1) was also built, and the results are provided in Figure S5 of the Supplementary Materials. However, no clear clustering among honey samples can be observed based on their geographical region of production. This result was attributed to the difficulty of really delimiting geographical regions when a natural product produced by bees is addressed. To try to simplify the analytical problem, three main Spanish geographical regions (related with different climatic conditions) were considered: the Cantabrian Sea region (North of Spain), the Mediterranean Sea region (east of Spain), and the Continental region (landlocked inland regions). The PLS-DA model obtained when these three regions were considered is depicted in Figure S6 (Supplementary Materials), as can be seen, sample discrimination was not accomplished.

Thus, the proposed non-targeted HPLC-UV chromatographic fingerprints are good sample chemical descriptors to authenticate and discriminate between blossom and honeydew honeys, as well as within several botanical varieties within blossom honeys. Unfortunately, no discrimination between honeydew honeys nor between the sample geographical region of production was achieved.

### 3.2. Off-Line SPE HPLC-UV Polyphenolic Fingerprinting Methodology

As polyphenolic compounds are among the most important bioactive substances found in honey, their presence and distribution can also be employed as sample markers to assess their classification and authentication. With this in mind, another aim of the present contribution was to develop an HPLC-UV fingerprinting methodology optimized for the isolation and detection of polyphenolic compounds. Under this approach, no compound quantification was intended but just the chromatographic signals were exploited as the data to take advantage of the fingerprinting approach. Hence, an off-line SPE method for the isolation and preconcentration of selected polyphenols was designed as honey sample treatment and the resulting extracts were analyzed by a reversed-phase HPLC-UV method optimized for the separation of selected polyphenols.

#### 3.2.1. Optimization of the Chromatographic Separation

**Figure 4:**
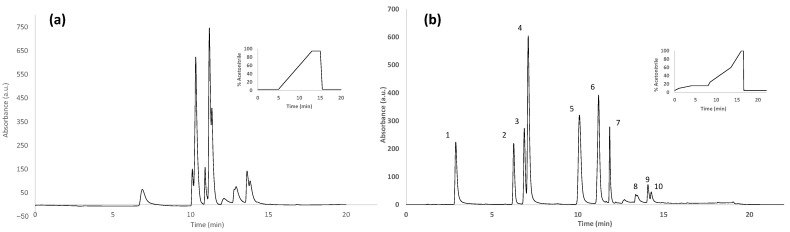
HPLC-UV chromatograms (at 280 nm) for a mixture of 10 selected polyphenols (at 25 mg L^−1^- each) obtained under (**a**) a universal gradient elution and (**b**) the proposed optimized gradient elution. Gradient programs are also depicted in the figures. Peak identification: 1, gallic acid; 2, *p*-hydroxybenzoic acid; 3, vanillic acid; 4, caffeic acid; 5, *p*-coumaric acid; 6, ferulic acid; 7, hesperidin; 8, luteolin; 9, apigenin; 10, kaempferol.

Taking into consideration the wide variability of polyphenolic compounds that can be present in the analyzed honey samples depending on their botanical variety as well as other factors such as the geographical region of production, environmental conditions, etc., the chromatographic separation of the HPLC-UV polyphenolic fingerprinting methodology was optimized based on 10 selected polyphenols belonging to different families. As normally described in the literature for the separation of polyphenols [40], a C18 reversed-phase column was employed working under gradient elution conditions generated with 0.1% formic acid aqueous solution and acetonitrile as mobile phase components. As a first attempt, a universal gradient program was used (the same as previously proposed for the non-targeted HPLC-UV method), which consisted of a 5 min isocratic elution at 3% acetonitrile and then increasing the organic content from 3% to 95% in 8 min. Under these conditions, most of the targeted polyphenolic compounds eluted in the chromatographic range from 10 to 15 min, with important coelutions among them, as can be seen in Figure 4a.

The chromatographic separation was then optimized by combining gradient and isocratic elution steps to attain a high chromatographic resolution for the studied polyphenols within a low analysis time. Figure 4b shows the chromatogram of a mixture of the 10 selected polyphenols under the proposed optimal separation conditions, where almost all the studied polyphenolic compounds are baseline separated.

#### 3.2.2. Optimization of the Off-Line SPE Extraction of Polyphenols in Honey Samples

The extraction of polyphenolic compounds from the analyzed honey samples relied on an off-line solid-phase extraction procedure using Oasis HLB (3 cc, 60 mg) cartridges. The optimization of the SPE procedure was based on the recovery of six selected polyphenols (gallic acid, *p*-hydroxybenzoic acid, vanillic acid, *p*-coumaric acid, apigenin, and kaempferol), from a heather honey sample. For such a purpose, a blank heather honey sample and a fortified heather honey sample at 25 mg kg**^−^**^1^ with the six selected polyphenols were analyzed. As a first experiment, 1 g of each heather honey sample was dissolved in 10 mL of acidified water (pH 2.0 with HCl) and loaded into the HLB SPE cartridge after conditioning with methanol and acidified water. Then, retained analytes were eluted with 1 mL of methanol, evaporated to dryness under nitrogen stream and in a water bath at 40 °C, and then reconstituted in 1 mL of water:acetonitrile (95:5 *v*/*v*). The extracts were then analyzed with the HPLC-UV polyphenolic method previously optimized. A standard solution of the six selected polyphenols at a concentration of 25 mgL**^−^**^1^ (equivalent to a 100% recovery from the fortified honey sample) was also analyzed. The recovery for each evaluated polyphenol in the heather honey sample was then calculated using the peak areas (A) as follows:

% Recovery = ((Afortified honey − Ahoney blank)/Astandard 100% recovery) × 100

Under these conditions, very different recovery values were obtained depending on the polyphenolic nature. Recoveries higher than 50% were observed for phenolic acids such as gallic acid (82%), *p*-hydroxybenzoic acid (67%), vanillic acid (54%), and *p*-coumaric acid (81%). In contrast, flavonoid compounds were poorly extracted, with values such as 6.2% and 3.4% for apigenin and kaempferol, respectively. These two flavonoids were not detected in the sample eluate after sample loading into the HLB SPE cartridges, confirming that the compounds were perfectly retained into the employed cartridges but not eluted under the tested conditions (1 mL methanol as elution solvent). Thus, polyphenolic elution was optimized by employing both methanol and acetonitrile as solvents, with the aim of evaluating the influence of eluotropic strengths as well as the elution volume (2, 4, and 6 mL). All the experiments were performed in triplicate, and the obtained recoveries are summarized in Table 2.

As can be seen, in general methanol provided higher recoveries than acetonitrile, thus resulting in a better solvent to elute the preconcentrated polyphenols from the HLB SPE cartridges. Recoveries obtained with methanol as elution solvent improved, in general, with the elution volume, thus 6 mL methanol were selected as the optimal elution volume for the proposed off-line SPE methodology.

#### 3.2.3. Analysis of Honey Samples with the Off-Line SPE HPLC-UV Polyphenolic Fingerprinting Methodology

**Figure 5:**
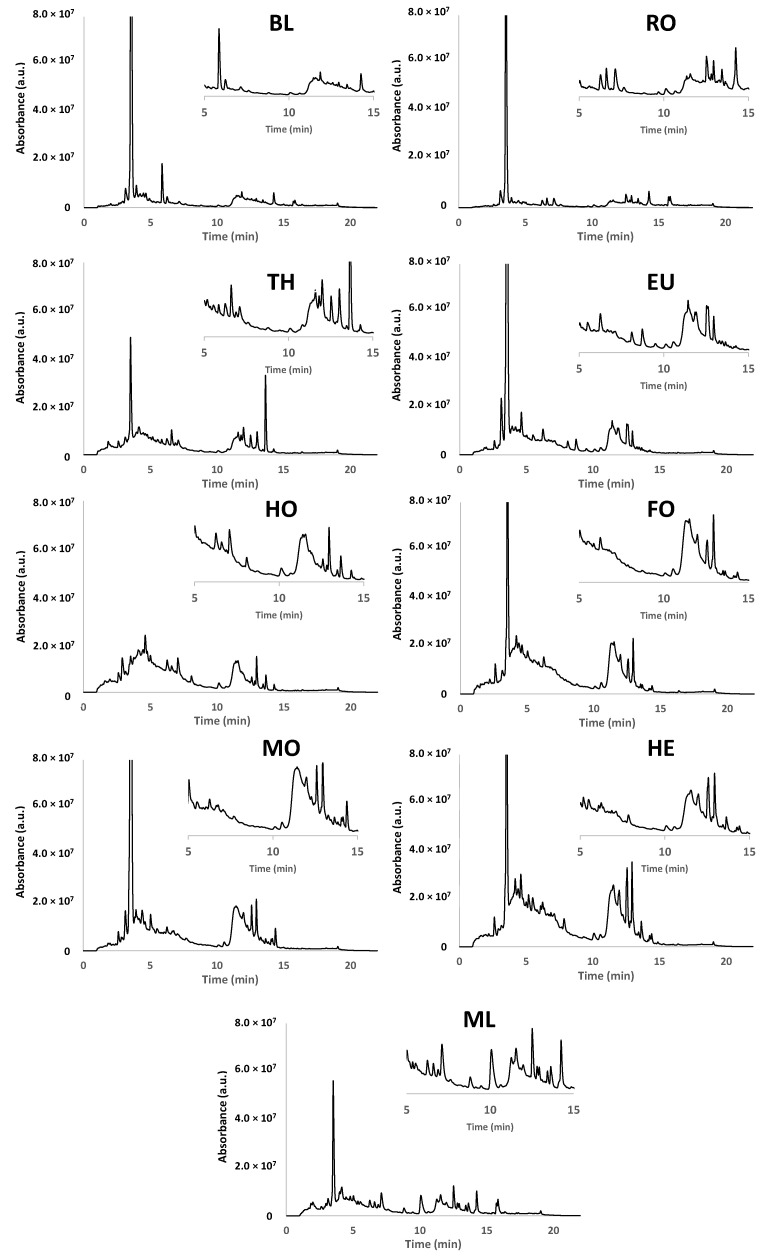
Off-line SPE HPLC-UV polyphenolic chromatographic fingerprints at 280 nm for one selected blossom and honeydew honey sample of each botanical origin under study. The time window from 5 to 15 min is enlarged in each inset. BL: Orange/lemon blossom; EU: Eucalyptus; FO: Forest; HE: Heather; HO: Holm oak; MF: Multifloral; MO: Mountain; RO: Rosemary; TH: Thyme.

The optimized off-line SPE HPLC-UV polyphenolic method was then employed for the analysis of the 136 honey samples under study. Chromatograms for one selected honey sample of each variety are depicted in Figure 5. These fingerprints are richer in signals than those obtained by the previously commented on non-targeted HPLC-UV chromatographic fingerprinting method (Figure 1). As an example, in Figure S7 (Supplementary Material) the fingerprints obtained for a multifloral honey sample with the two methods are overlapped. As commented, the off-line SPE HPLC-UV fingerprints are richer in signals, belonging some of them to the polyphenolic family, although other bioactive substance families could have been isolated, preconcentrated and detected under these working conditions. In any case and taking into consideration the aim of fingerprinting approaches, it is not necessary to know the identity of the detected compounds as all the registered instrumental signals will be employed as sample chemical descriptors to assess sample characterization, classification, and authentication.

As observed in Figure 5, the off-line SPE HPLC-UV fingerprints are characterized by two featured regions (regions from 1 to 10 min, and from 10 to 15 min), with the presence of multiple peak signals of different intensities. With these fingerprints and by visual inspection it is not easy to find completely similar patterns among the analyzed honey samples as in the case of the non-targeted HPLC-UV fingerprints described in Figure 1. However, some important differences can be observed. For example, an important peak signal, quite intense in most of the analyzed honey samples, is observed at a retention time around 3.5 min, which is not present at all in holm oak honey samples. Remarkable differences can also be observed in the region from 10 to 15 min among almost all the analyzed honey samples regarding both peak signals and intensities, highlighting orange/lemon blossom honey samples which do not depict many signals in this chromatographic time window. In contrast, they present a relatively high peak signal at a retention time around 6 min, almost not present in any of the other honey samples. Finally, multifloral honeys are also characterized by a wide peak signal at a retention time around 10 min which seems to be also present in other honey samples such as heather and holm oak.

Even though no similar patterns can be found visually, the obtained off-line SPE HPLC-UV fingerprints were again quite reproducible within the same honey botanical origin, so they were also evaluated as sample chemical descriptors to characterize and classify the analyzed honey samples.

#### 3.2.4. Chemometric Data Analysis

First, a PCA was applied to the off-line SPE HPLC-UV fingerprints as honey sample chemical descriptors, and the scatter plot of scores of PC1 vs. PC2 is depicted in Figure S8 (Supplementary Material). As can be observed, QCs appeared again completely clustered in the center of the plot demonstrating the reproducibility and the robustness of the proposed HPLC-UV methodology and that chemometric results are not affected by instrumental drifts. As regards sample distribution, MF honeys are distributed through all the score plot because of their higher variability in botanical compositions. The other honey samples tend to be grouped according to their botanical origin but, as in the case of the non-targeted HPLC-UV fingerprints, three main grouping regions can be discriminated through PC1: (i) honeydew honeys (MO, FO, and HO) and heather blossom honey samples were clustered exhibiting positive PC1 values and widely distributed through PC2 (except for HO samples, where all of them depicted negative PC2 values); (ii) blossom thyme and eucalyptus honey samples, which were clustered in the central area of the plot; and (iii) orange/lemon blossom and rosemary honey samples, which appeared at the left area of the plot, with negative PC1 values and distributed through PC2. Therefore, similar distribution by the PCA to the one obtained by non-targeted HPLC-UV fingerprinting was achieved.

**Figure 6:**
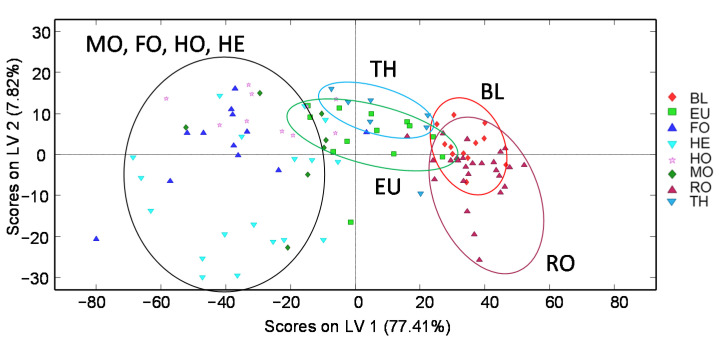
PLS-DA score plot of LV1 vs. LV2 when using off-line SPE HPLC-UV polyphenolic fingerprints (at 280 nm) as honey chemical descriptors for botanical origin classification (4 LVs were used to build the model). QCs and multifloral honeys were excluded. BL: Orange/lemon blossom; EU: Eucalyptus; FO: Forest; HE: Heather; HO: Holm oak; MO: Mountain; RO: Rosemary; TH: Thyme.

The off-line SPE HPLC-UV fingerprints were also subjected to a PLS-DA for sample characterization and classification based on the honey botanical origin, and the score plot of LV1 vs. LV2 is shown in Figure 6. To simplify the model, QCs and multifloral honeys (which appeared dispersed through all the score plot) were not considered. As can be seen in the figure, honey samples are discriminated through LV1 in the three main groups previously commented, although a higher discrimination capacity was encountered in comparison to previously described models. Orange/lemon blossom and rosemary blossom honeys are located at the right part of the plot (presenting positive LV1 values) but almost completely discriminated from RO samples by LV2. BL samples tend to present positive LV2 values while most of RO samples exhibit negative LV2 values. The group of thyme and eucalyptus blossom honeys, which are in the center part of the plot, mainly exhibit positive LV2 values, being higher, in general, for the thyme honeys. Finally, the third group consisted of honeydew honeys and the heather blossom honeys, which are distributed in the left area of the plot (with negative LV1 values). HE honeys are the more dispersed samples, while holm oak honeydew honeys are grouped in the top-left area of the plot (negative LV1 and positive LV2 values).

A paired PLS-DA model based on blossom honeys against honeydew honeys was also validated to evaluate the classification capacity of the proposed off-line SPE HPLC-UV polyphenolic fingerprints. For that purpose, 70% of the samples were randomly selected as the calibration set and the remaining 30% of the samples used as prediction set, and the results are shown in Figure S9 (Supplementary Materials). Considering the variability within the analyzed sample, acceptable results were obtained showing sensitivity values of 0.825, 0.825, and 0.895, and specificity values of 0.824, 0.824, and 0.750, for calibration, cross-validation, and prediction, respectively. Thus, classification errors of 18% and 16% for calibration and prediction, respectively, were observed.

**Figure 7:**
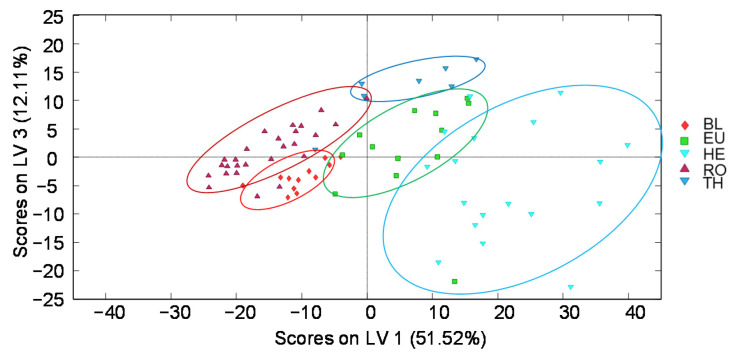
PLS-DA score plot of LV1 vs. LV3 when using off-line SPE HPLC-UV polyphenolic fingerprints (at 280 nm) as honey chemical descriptors of blossom honeys for botanical origin classification (4 LVs were used to build the model). BL: Orange/lemon blossom; EU: Eucalyptus; HE: Heather; RO: Rosemary; TH: Thyme.

PLS-DA models considering honeydew or blossom honeys independently, were also studied by employing off-line SPE HPLC-UV fingerprints as chemical descriptors. Noticeable results were obtained when focusing only on blossom honeys as depicted in Figure 7 (PLS-DA score plot of LV1 vs. LV3). The samples are clustered according to their botanical origin and very acceptable discrimination among the five sample groups was achieved, obtaining a much better sample classification than the one observed when using non-targeted HPLC-UV fingerprints as sample chemical descriptors (Figure S3 in Supplementary Material). In contrast, sample discrimination was hindered when addressing only the classification of honeydew honeys (Figure S10 in Supplementary Material), as previously observed (Figure S4 in Supplementary Material).

Despite the overlapping of some sample groups, the discrimination capacity clearly improved when employing the PLS-DA pair models using off-line SPE HPLC-UV fingerprints, even for those honey groups that are clustered together in previous commented models. This can be clearly observed in the PLS-DA pair models of BL vs. RO, TH vs. EU, HO vs. MO, and HO vs. FO in Figure S11 (Supplementary Material). This level of discrimination was not accomplished for some of these pairs when using non-targeted HPLC-UV fingerprints, demonstrating the higher discrimination power of the off-line SPE HPLC-UV fingerprints for the characterization and classification of honey samples based on their botanical origin.

A multiclass model was built specifically to assign the set of blossom honey samples, including BL, EU, HE, RO, and TF classes simultaneously. Table 3 provides the sensitivity, specificity and overall class prediction error assessed according to cross-validation. It should be mentioned that, since the performance of the classification model including honeydew samples was more limited, with sensitivity and specificity values often below 80%, these types of samples was excluded from the multiclass model. In general, values were highly satisfactory, with sensitivities and specificities better that 90% and percentages of wrongly assigned samples, including false negatives and false positives below 5%. In the same way, the confusion matrix was [11 1 0 0 0; 0 12 1 0 0; 0 0 18 0 0; 0 0 0 25 1; 0 0 0 2 5], with row/columns corresponding to the number of actual/predicted BL, EU, HE, RO, and TH, respectively. Hence, it was found that HE was the most heterogeneous and different class and some confusion among RO and TF was detected.

Finally, off-line SPE HPLC-UV fingerprints were also evaluated for the classification of the analyzed honey samples based on honey geographical origin by the PLS-DA. Nevertheless, neither when considering all the Spanish geographical production regions under study (see Table 1), nor when considering geographical regions related to different climatic conditions, acceptable sample discrimination was accomplished, obtaining similar results to the ones previously described when using non-targeted HPLC-UV fingerprints.

Thus, the off-line SPE HPLC-UV chromatographic fingerprints are also good sample chemical descriptors to authenticate and discriminate between blossom honeys and honeydew honeys, as well as within blossom honey varieties and some honeydew honey samples. The overall performance was enhanced with respect to non-targeted HPLC-UV chromatographic fingerprinting but no discrimination regarding the sample geographical region of production was achieved.

## 4. Conclusions

In the present work, non-targeted HPLC-UV and off-line SPE HPLC-UV fingerprints have proved to be useful sample chemical descriptors for the characterization, classification, and authentication of blossom and honeydew honeys according to their botanical origin. The non-targeted HPLC-UV fingerprints were easily obtained after a simple honey dilution and reversed-phase chromatography using a universal gradient elution. In the case of the polyphenolic fingerprinting method, an off-line SPE procedure using HLB cartridges was developed, providing very acceptable recoveries, with values higher than 67% for all the polyphenols studied. A gradient elution program was designed for the separation and detection of polyphenols, giving place to richer fingerprints in both peak signals and intensities in comparison to those observed with the non-targeted HPLC-UV method.

The exploratory analysis by a PCA and a PLS-DA for both non-targeted HPLC-UV and off-line SPE HPLC-UV fingerprints showed, in general, good discrimination capabilities among the different analyzed blossom and honeydew honey samples based on the botanical origin. Specifically, three main groups were clearly distinguished: orange/lemon blossom and rosemary honeys; thyme and eucalyptus honeys; and heather blossom honey and holm oak, mountain, and forest honeydew honeys. Heather blossom honey appeared always clustered together with honeydew honeys because of their common physicochemical and organoleptic characteristics, as described in the literature.

Off-line HPLC-UV fingerprints exhibited superior honey classification capabilities in comparison to non-targeted HPLC-UV fingerprints as, for example, discrimination among the five blossom honey botanical origins under study (orange/lemon blossom, rosemary, thyme, eucalyptus, and heather) was also accomplished.

Therefore, the proposed HPLC-UV fingerprinting methodologies resulted to be reliable and cheap methodologies to address honey characterization and authentication based on botanical origins.

## Figures and Tables

**Table 1:** Analyzed honey samples considering botanical and geographical origin.

Honey	Botanical Variety	Number of Samples	Geographical Origin	Number of Samples
Blossom honeys	Orange/Lemon Blossom (BL)	12	Aragon	1
			Balearic Islands	2
			Cantabria	1
			Castile and Leon	2
			Catalonia	3
			Extremadura	1
			Spain ^a^	2
	Eucalyptus (EU)	13	Andalusia	1
			Asturias	2
			Cantabria	1
			Castile and Leon	2
			Catalonia	1
			Extremadura	4
			Navarre	1
			Spain and others ^b^	1
	Rosemary (RO)	26	Andalusia	1
			Aragon	4
			Asturias	1
			Balearic Islands	4
			Castile and Leon	1
			Cantabria	2
			Catalonia	6
			Extremadura	4
			Navarre	1
			Spain ^a^	2
	Thyme (TH)	7	Castile and Leon	1
			Castile La Mancha	2
			Catalonia	1
			Extremadura	2
			Spain ^a^	1
	Heather (HE)	18	Asturias	2
			Basque Country	1
			Cantabria	3
			Castile and Leon	5
			Catalonia	2
			Extremadura	5
Honeydew honeys	Mountain (MO)	6	Asturias	2
			Castile and Leon	1
			Castile La Mancha	1
			Catalonia	2
	Forest (FO)	10	Balearic Islands	2
			Cantabria	2
			Castile and Leon	1
			Catalonia	1
			Spain ^a^	4
	Holm Oak (HO)	10	Aragon	2
			Castile and Leon	2
			Extremadura	6
Other honeys	Multifloral (MF)	34	Asturias	1
			Balearic Islands	4
			Cantabria	1
			Castile and Leon	7
			Castile La Mancha	2
			Catalonia	8
			Extremadura	4
			Navarre	4
			Spain ^a^	1
			Spain and others ^b^	2

^a^ Spain: honeys produced in Spain but geographical region not specified; ^b^ Spain and others: honeys that include mixtures of honey produced in Spain and other countries such as Uruguay, Cuba, Mexico, Romania or Ukraine.

**Table 2:** Off-line SPE recovery values for 6 selected polyphenolic compounds when employing different elution solvents and volumes.

Compound	Recoveries (%) ^a^ (Standard Deviation Provided within Parenthesis)					
	Methanol			Acetonitrile		
	2 mL	4 mL	6 mL	2 mL	4 mL	6 mL
gallic acid	82 (7)	83 (4)	72 (9)	1 (2)	14 (16)	14 (13)
*p*-hydroxybenzoic acid	93 (4)	110 (10)	97 (12)	46 (8)	81 (7)	110 (8)
vanillic acid	72 (1)	74 (2)	67 (5)	77 (1)	73 (1)	87 (8)
*p*-coumaric acid	87 (2)	87.3 (0.9)	84 (6)	42 (11)	77 (2)	90 (5)
apigenin	57 (3)	50 (11)	110 (8)	0.2 (0.1)	0 (0)	2 (4)
kaempferol	52 (4)	112 (12)	142 (22)	7 (8)	1 (2)	60 (22)

^a^ *n* = 3.

**Table 3:** Cross-validated multiclass predictions for the set of blossom honey samples using 8 latent variables. BL: Orange/lemon blossom; EU: Eucalyptus; HE: Heather; RO: Rosemary; TH: Thyme.

Class	Sensitivity (%)	Specificity (%)	Classification Error (%)
BL	91.7	98.4	2.6
EU	92.3	90.5	7.9
HE	100	94.8	3.9
RO	96.2	94.0	3.9
TH	71.4	89.9	9.2

## Data Availability

Data is available upon request to the authors.

## Supplementary Materials

The following supporting information can be downloaded at: https://www.mdpi.com/article/10.3390/foods11152345/s1, Figure S1: X-data matrix used for PCA and PLS-DA; Figure S2: Validation of a paired PLS-DA model of blossom honeys versus honeydew honeys when using non-targeted HPLC-UV fingerprints as sample chemical descriptors. Red line establishes the separation between both classes. Filled symbols correspond to the calibration set and empty symbols correspond to the validation set (unknown samples for prediction purposes); Figure S3: Supervised PLS-DA score plots of LV1 vs. LV2 when using non-targeted HPLC-UV chromatographic fingerprints (at 280 nm) as chemical descriptors of blossom honeys for botanical origin classification (2 LVs were used to build the model). BL: Orange/lemon blossom; EU: Eucalyptus; HE: Heather; RO: Rosemary; TH: Thyme.; Figure S4: Supervised PLS-DA score plots of LV1 vs. LV2 when using non-targeted HPLC-UV chromatographic fingerprints (at 280 nm) as chemical descriptors of honeydew honeys for botanical origin classification (2 LVs were used to build the model). FO: Forest; HO: Holm oak; MO; Mountain; Figure S5: Supervised PLS-DA score plots of LV1 vs. LV2 when using non-targeted HPLC-UV chromatographic fingerprints (at 280 nm) as honey chemical descriptors for geographical origin classification (4 LVs were used to build the model). QCs were not considered. AN: Andalusia; AR: Aragon; AS: Asturias; BC: Basque Country; CN: Cantabria; CM: Castile La Mancha; CL: Castile and Leon; CT: Catalonia; E: Extremadura; BI: Balearic Islands; N: Navarre; S: Spain; SO: Spain and others; Figure S6: Supervised PLS-DA score plots of LV1 vs. LV2 when using non-targeted HPLC-UV chromatographic fingerprints (at 280 nm) as honey chemical descriptors for geographical origin classification related with different weather conditions (2 LVs were used to build the model). QCs were not considered; Figure S7: HPLC-UV chromatograms (at 280 nm) obtained for a selected multifloral honey sample. (a) non-targeted HPLC-UV fingerprinting method; (b) off-line SPE HPLC-UV polyphenolic fingerprinting method; Figure S8: PCA score plot of PC1 vs. PC2 (a) and plot of loadings of PC1 and PC2 (b) when using off-line SPE HPLC-UV polyphenolic fingerprints (at 280 nm) as honey chemical descriptors (3 PCs were used to build the model); Figure S9: Validation of a paired PLS-DA model of blossom honeys versus honeydew honeys when using off-line SPE HPLC-UV polyphenolic fingerprints as sample chemical descriptors. Red line establishes the separation between both classes. Filled symbols correspond to the calibration set and empty symbols correspond to the validation set (unknown samples for prediction purposes). Figure S10: Supervised PLS-DA score plots of LV1 vs. LV2 when using off-line SPE HPLC-UV polyphenolic fingerprints (at 280 nm) as honey chemical descriptors of honeydew honeys for botanical origin classification (2 LVs were used to build the model). FO: Forest; HO: Holm oak; MO; Mountain; Figure S11: Supervised PLS-DA score plots of LV1 vs. LV2 when using off-line SPE HPLC-UV polyphenolic fingerprints (at 280 nm) as honey chemical descriptors of (a) orange/lemon blossom vs. rosemary blossom honeys (3 LVs were used to build the model), (b) thyme vs. eucalyptus blossom honeys (2 LVs were used to build the model), (c) holm oak vs. mountain honeydew honeys (2 LVs were used to build the model), and (d) holm oak vs. forest honeydew honeys (2 LVs were used to build the model). BL: Orange/lemon blossom; EU: Eucalyptus; FO: Forest; HO: Holm oak; MO; Mountain; RO: Rosemary; TH: Thyme.

## References

[1] Da Silva P.M., Gauche C., Gonzaga L.V., Costa A.C.O., Fett R. (2016). Honey: Chemical composition, stability and authenticity. Food Chem..

[2] Seraglio S.K.T., Silva B., Bergamo G., Brugnerotto P., Gonzaga L.V., Fett R., Costa A.C.O. (2019). An overview of physicochemical characteristics and health-promoting properties of honeydew honey. Food Res. Int..

[3] Pita-Calvo C., Vázquez M. (2017). Differences between honeydew and blossom honeys: A review. Trends Food Sci. Technol..

[4] Recklies K., Peukert C., Kölling-Speer I., Speer K. (2021). Differentiation of Honeydew Honeys from Blossom Honeys and According to Their Botanical Origin by Electrical Conductivity and Phenolic and Sugar Spectra. J. Agric. Food Chem..

[5] (2002). Council Directive 2001/110/EC of 20 December 2001 relating to honey. Off. J. Eur. Communities.

[6] Pita-Calvo C., Vázquez M. (2018). Honeydew Honeys: A Review on the Characterization and Authentication of Botanical and Geographical Origins. J. Agric. Food Chem..

[7] Consonni R., Cagliani L.R. (2015). Recent developments in honey characterization. RSC Adv..

[8] Wang S., Guo Q., Wang L., Lin L., Shi H., Cao H., Cao B. (2015). Detection of honey adulteration with starch syrup by high performance liquid chromatography. Food Chem..

[9] Wu L., Du B., Vander Heyden Y., Chen L., Zhao L., Wang M., Xue X. (2017). Recent advancements in detecting sugar-based adulterants in honey—A challenge. TrAC—Trends Anal. Chem..

[10] Du B., Wu L., Xue X., Chen L., Li Y., Zhao J., Cao W. (2015). Rapid Screening of Multiclass Syrup Adulterants in Honey by Ultrahigh-Performance Liquid Chromatography/Quadrupole Time of Flight Mass Spectrometry. J. Agric. Food Chem..

[11] Tsagkaris A.S., Koulis G.A., Danezis G.P., Martakos I., Dasenaki M., Georgiou C.A., Thomaidis N.S. (2021). Honey authenticity: Analytical techniques, state of the art and challenges. RSC Adv..

[12] Kaškoniene V., Venskutonis P.R. (2010). Floral Markers in Honey of Various Botanical and Geographic Origins: A Review. Compr. Rev. Food Sci. Food Saf..

[13] Wang X., Chen Y., Hu Y., Zhou J., Chen L., Lu X. (2022). Systematic Review of the Characteristic Markers in Honey of Various Botanical, Geographic, and Entomological Origins. ACS Food Sci. Technol..

[14] Sergiel I., Pohl P., Biesaga M. (2014). Characterisation of honeys according to their content of phenolic compounds using high performance liquid chromatography/tandem mass spectrometry. Food Chem..

[15] Koulis G.A., Tsagkaris A.S., Aalizadeh R., Dasenaki M.E., Panagopoulou E.I., Drivelos S., Halagarda M., Georgiou C.A., Proestos C., Thomaidis N.S. (2021). Honey phenolic compound profiling and authenticity assessment using hrms targeted and untargeted metabolomics. Molecules.

[16] Campone L., Piccinelli A.L., Pagano I., Carabetta S., Di Sanzo R., Russo M., Rastrelli L. (2014). Determination of phenolic compounds in honey using dispersive liquid-liquid microextraction. J. Chromatogr. A.

[17] Kawashima H., Suto M., Suto N. (2018). Determination of carbon isotope ratios for honey samples by means of a liquid chromatography/isotope ratio mass spectrometry system coupled with a post-column pump. Rapid Commun. Mass Spectrom..

[18] Ciucure C.T., Geană E.I. (2019). Phenolic compounds profile and biochemical properties of honeys in relationship to the honey floral sources. Phytochem. Anal..

[19] Campillo N., Viñas P., Férez-Melgarejo G., Hernández-Córdoba M. (2015). Dispersive liquid-liquid microextraction for the determination of flavonoid aglycone compounds in honey using liquid chromatography with diode array detection and time-of-flight mass spectrometry. Talanta.

[20] Zhou J., Yao L., Li Y., Chen L., Wu L., Zhao J. (2014). Floral classification of honey using liquid chromatography-diode array detection-tandem mass spectrometry and chemometric analysis. Food Chem..

[21] Dong H., Luo D., Xian Y., Luo H., Guo X., Li C., Zhao M. (2016). Adulteration Identification of Commercial Honey with the C-4 Sugar Content of Negative Values by an Elemental Analyzer and Liquid Chromatography Coupled to Isotope Ratio Mass Spectroscopy. J. Agric. Food Chem..

[22] Wang X., Rogers K.M., Li Y., Yang S., Chen L., Zhou J. (2019). Untargeted and Targeted Discrimination of Honey Collected by Apis cerana and Apis mellifera Based on Volatiles Using HS-GC-IMS and HS-SPME-GC-MS. J. Agric. Food Chem..

[23] Spiteri M., Rogers K.M., Jamin E., Thomas F., Guyader S., Lees M., Rutledge D.N. (2017). Combination of 1H NMR and chemometrics to discriminate manuka honey from other floral honey types from Oceania. Food Chem..

[24] Zhang J., Chen H., Fan C., Gao S., Zhang Z., Bo L. (2020). Classification of the botanical and geographical origins of Chinese honey based on 1H NMR profile with chemometrics. Food Res. Int..

[25] Li S., Zhang X., Shan Y., Su D., Ma Q., Wen R., Li J. (2017). Qualitative and quantitative detection of honey adulterated with high-fructose corn syrup and maltose syrup by using near-infrared spectroscopy. Food Chem..

[26] Latorre C.H., Crecente R.M.P., Martín S.G., García J.B. (2013). A fast chemometric procedure based on NIR data for authentication of honey with protected geographical indication. Food Chem..

[27] Guelpa A., Marini F., du Plessis A., Slabbert R., Manley M. (2017). Verification of authenticity and fraud detection in South African honey using NIR spectroscopy. Food Control.

[28] Valinger D., Longin L., Grbeš F., Benković M., Jurina T., Gajdoš Kljusurić J., Jurinjak Tušek A. (2021). Detection of honey adulteration—The potential of UV-VIS and NIR spectroscopy coupled with multivariate analysis. LWT.

[29] Lenhardt L., Bro R., Zeković I., Dramićanin T., Dramićanin M.D. (2015). Fluorescence spectroscopy coupled with PARAFAC and PLS DA for characterization and classification of honey. Food Chem..

[30] Ansari M.J., Al-Ghamdi A., Khan K.A., Adgaba N., El-Ahmady S.H., Gad H.A., Roshan A., Meo S.A., Kolyali S. (2018). Validation of botanical origins and geographical sources of some Saudi honeys using ultraviolet spectroscopy and chemometric analysis. Saudi J. Biol. Sci..

[31] Suhandy D., Yulia M. (2021). The use of UV spectroscopy and SIMCA for the authentication of Indonesian honeys according to botanical, entomological and geographical origins. Molecules.

[32] Cavazza A., Corradini C., Musci M., Salvadeo P. (2013). High-performance liquid chromatographic phenolic compound fingerprint for authenticity assessment of honey. J. Sci. Food Agric..

[33] Li Y., Jin Y., Yang S., Zhang W., Zhang J., Zhao W., Chen L., Wen Y., Zhang Y., Lu K. (2017). Strategy for comparative untargeted metabolomics reveals honey markers of different floral and geographic origins using ultrahigh-performance liquid chromatography-hybrid quadrupole-orbitrap mass spectrometry. J. Chromatogr. A.

[34] Machado A.M., Miguel M.G., Vilas-Boas M., Figueiredo A.C. (2020). Honey volatiles as a fingerprint for botanical origin—a review on their occurrence on monofloral honeys. Molecules.

[35] Sotiropoulou N.S., Xagoraris M., Revelou P.K., Kaparakou E., Kanakis C., Pappas C., Tarantilis P. (2021). The use of spme-gc-ms ir and raman techniques for botanical and geographical authentication and detection of adulteration of honey. Foods.

[36] Puscas A., Hosu A., Cimpoiu C. (2013). Application of a newly developed and validated high-performance thin-layer chromatographic method to control honey adulteration. J. Chromatogr. A.

[37] Massart D.L., Vandeginste B.G.M., Buydens L.M.C., de Jong S., Lewi P.J., Smeyers-Verbeke J. (1997). Handbook of Chemometrics and Qualimetrics. J. Chem. Inf. Comput. Sci..

[38] Al M.L., Daniel D., Moise A., Bobis O., Laslo L., Bogdanov S. (2009). Physico-chemical and bioactive properties of different floral origin honeys from Romania. Food Chem..

[39] Martos I., Cossentini M., Ferreres F., Tomás-Barberán F.A. (1997). Flavonoid Composition of Tunisian Honeys and Propolis. J. Agric. Food Chem..

[40] Lucci P., Saurina J., Núñez O. (2017). Trends in LC-MS and LC-HRMS analysis and characterization of polyphenols in food. TrAC—Trends Anal. Chem..

