# Supplementary material for: Characterization, Classification and Authentication of Spanish Blossom and Honeydew Honeys by Non-Targeted HPLC-UV and Off-Line SPE HPLC-UV Polyphenolic Fingerprinting Strategies
Source: Foods. 2022 Aug 5;11(15):2345. doi: 10.3390/foods11152345 (PMC9368295; doi:10.3390/foods11152345)
Supplement: Supplementary file 1 [file foods-11-02345-s001.zip › foods-1792526-supplementary.pdf]

Supplementary Material

Characterization, Classification and Authentication of Spanish Blossom and Honeydew Honeys by Non-Targeted HPLC-UV and Off-Line SPE HPLC-UV Polyphenolic Fingerprinting Strategies

Víctor García-Seval [1], Clàudia Martínez-Alfaro [1], Javier Saurina [1,2], Oscar Núñez [1,2,*] and Sònia Sentellas [1,2,3]

[1] Department of Chemical Engineering and Analytical Chemistry, Universitat de Barcelona, Martí i Franquès 1-11, E-08028 Barcelona, Spain; vgarciaseval@gmail.com (V.G.-S.); cmartinezialfaro@gmail.com (C.M.-A.); xavi.saurina@ub.edu (J.S.); sonia.sentellas@ub.edu (S.S.)
[2] Research Institute in Food Nutrition and Food Safety, Universitat de Barcelona, Recinte Torribera, Av. Prat de la Riba 171, Edifici de Recerca (Gaudí), Santa Coloma de Gramenet, E-08921 Barcelona, Spain
[3] Serra Húnter Fellow, Generalitat de Catalunya, Rambla de Catalunya 19-21, E-08007 Barcelona, Spain
* Correspondence: oscar.nunez@ub.edu

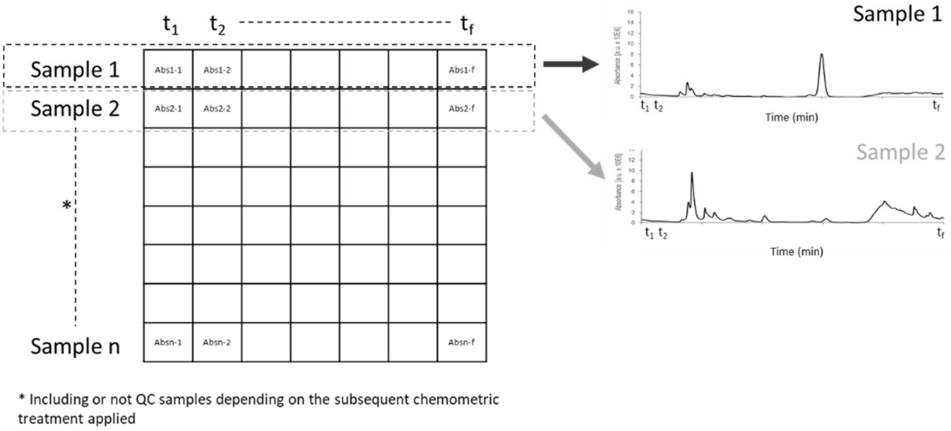

Figure S1. X-data matrix used for PCA and PLS-DA.

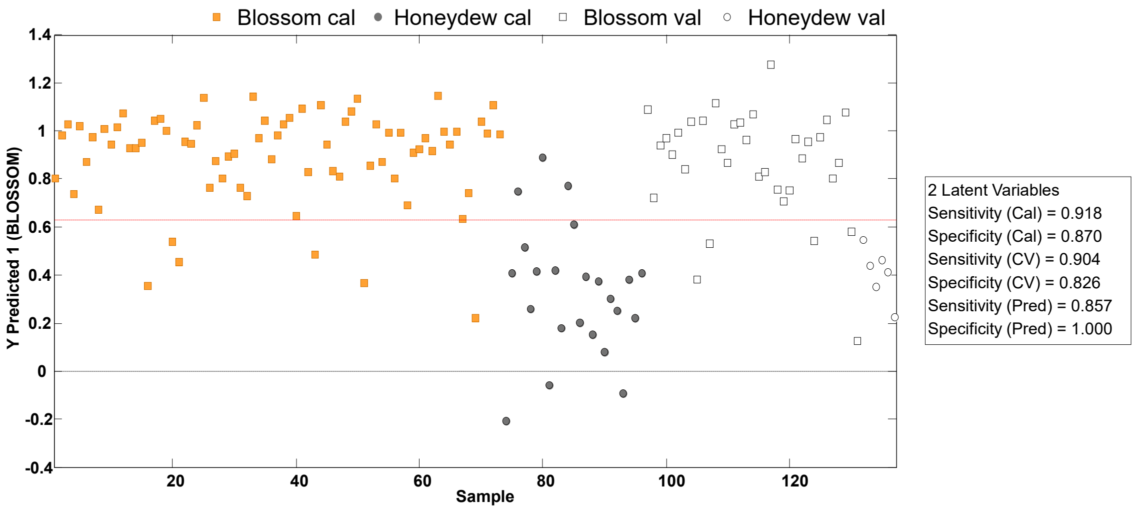

Figures S2. Validation of a paired PLS-DA model of blossom-honeys versus honeydew-honeys when using non-targeted HPLC-UV fingerprints as sample chemical descriptors. Red line establishes the separation between both classes. Filled symbols correspond to the calibration set and empty symbols correspond to the validation set (unknown samples for prediction purposes).

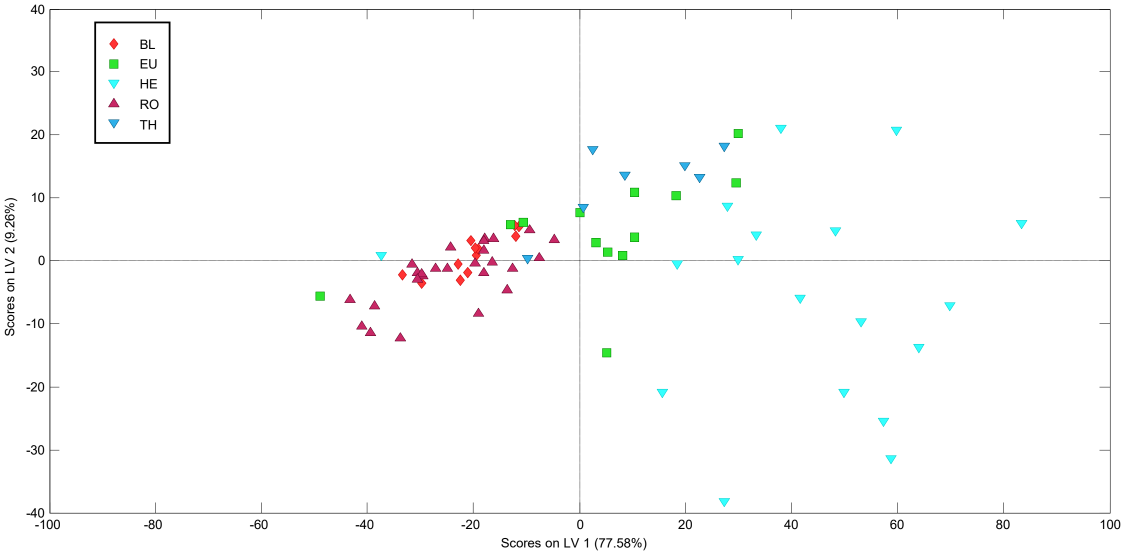

Figure S3. Supervised PLS-DA score plots of LV1 vs. LV2 when using non-targeted HPLC-UV chromatographic fingerprints (at 280 nm) as chemical descriptors of blossom honeys for botanical origin classification (2 LVs were used to build the model). BL: Orange/lemon blossom; EU: Eucalyptus; HE: Heather; RO: Rosemary; TH: Thyme.

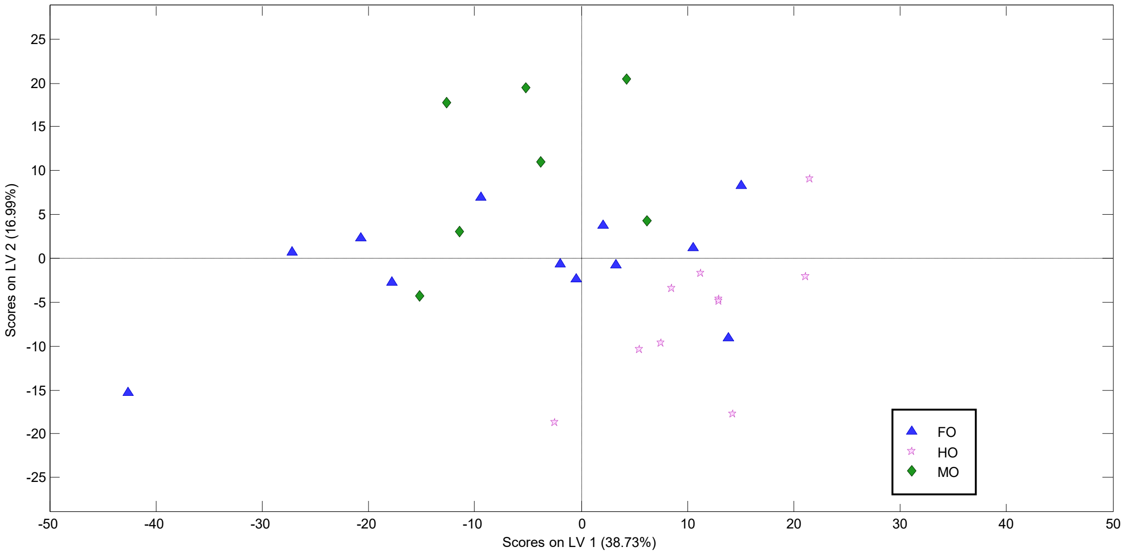

Figure S4. Supervised PLS-DA score plots of LV1 vs. LV2 when using non-targeted HPLC-UV chromatographic fingerprints (at 280 nm) as chemical descriptors of honeydew honeys for botanical origin classification (2 LVs were used to build the model). FO: Forest; HO: Holm oak; MO; Mountain.

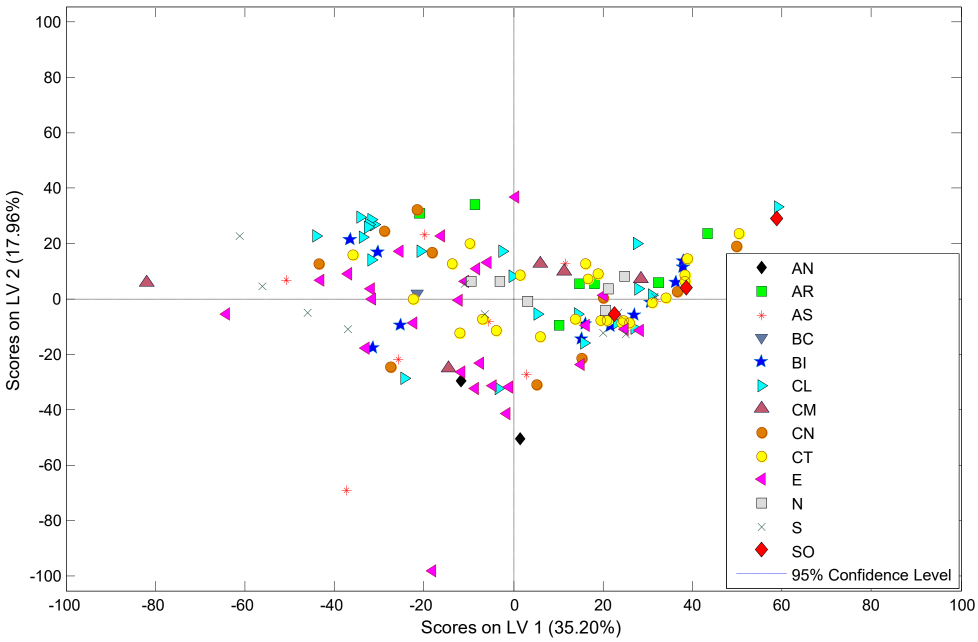

Figure S5. Supervised PLS-DA score plots of LV1 vs. LV2 when using non-targeted HPLC-UV chromatographic fingerprints (at 280 nm) as honey chemical descriptors for geographical origin classification (4 LVs were used to build the model). QCs were not considered AN: Andalusia; AR: Aragon; AS: Asturias; BC: Basque Country; CN: Cantabria; CM: Castile La Mancha; CL: Castile and Leon; CT: Catalonia; E: Extremadura; BI: Balearic Islands; N: Navarre; S: Spain; SO: Spain and others.

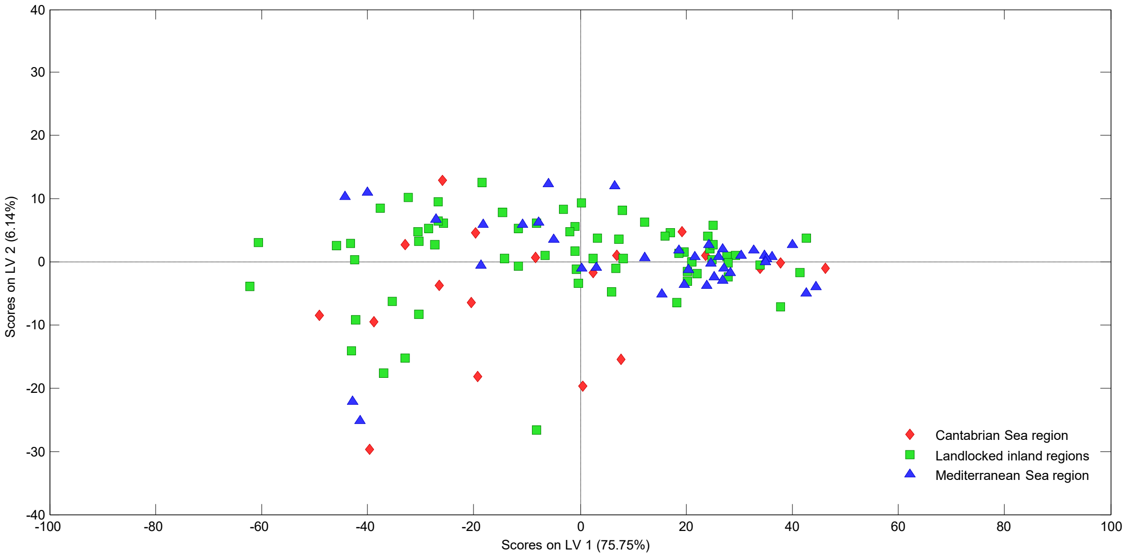

Figure S6. Supervised PLS-DA score plots of LV1 vs. LV2 when using non-targeted HPLC-UV chromatographic fingerprints (at 280 nm) as honey chemical descriptors for geographical origin classification related with different weather conditions (2 LVs were used to build the model). QCs were not considered.

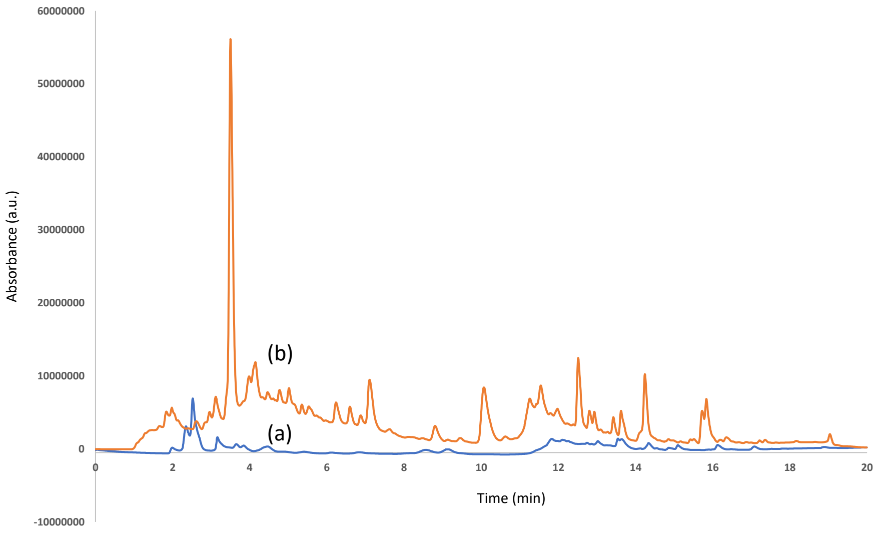

Figure S7. HPLC-UV chromatograms (at 280 nm) obtained for a selected multifloral honey sample. (a) non-targeted HPLC-UV fingerprinting method; (b) off-line SPE HPLC-UV fingerprinting method.

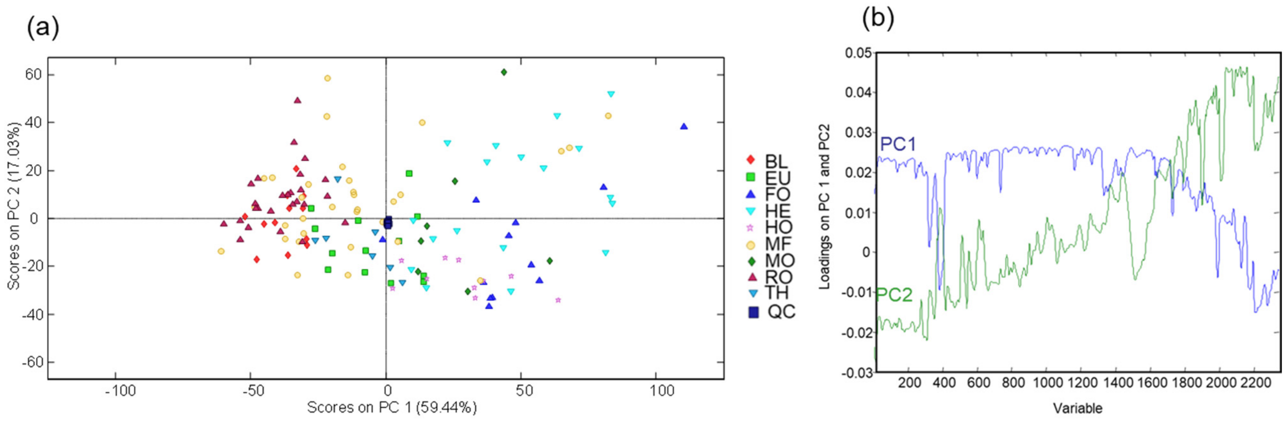

Figure S8. PCA score plot of PC1 vs PC2 (a) and plot of loadings of PC1 and PC2 (b) when using off-line SPE HPLC-UV fingerprints (at 280 nm) as honey chemical descriptors (3 PCs were used to build the model). BL: Orange/lemon blossom; EU: Eucalyptus; FO: Forest; HE: Heather; HO: Holm oak; MF; Multifloral; MO; Mountain; RO: Rosemary; TH: Thyme.

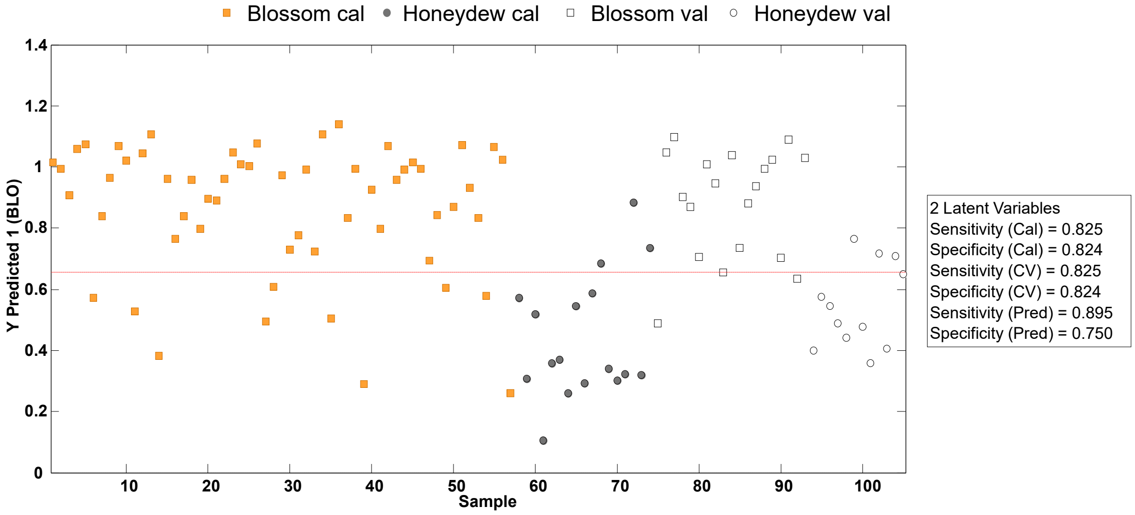

Figure S9. Validation of a paired PLS-DA model of blossom-honeys versus honeydew-honeys when using off-line SPE HPLC-UV polyphenolic fingerprints as sample chemical descriptors. Red line establishes the separation between both classes. Filled symbols correspond to the calibration set and empty symbols correspond to the validation set (unknown samples for prediction purposes).

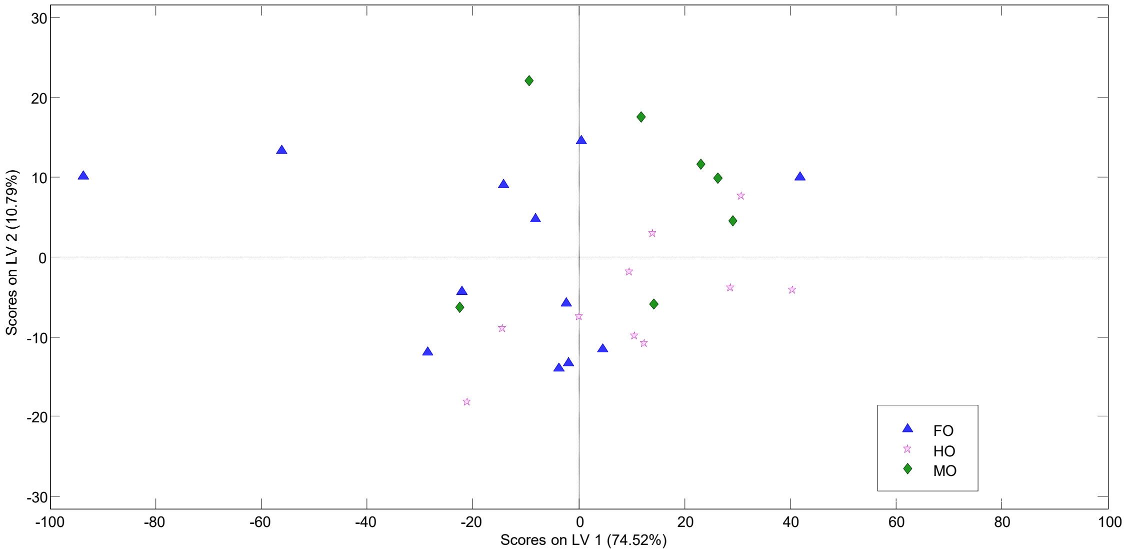

Figure S10. Supervised PLS-DA score plots of LV1 vs. LV2 when using off-line SPE HPLC-UV fingerprints (at 280 nm) as honey chemical descriptors of honeydew-honeys for botanical origin classification (2 LVs were used to build the modelFO: Forest; HO: Holm oak; MO; Mountain.

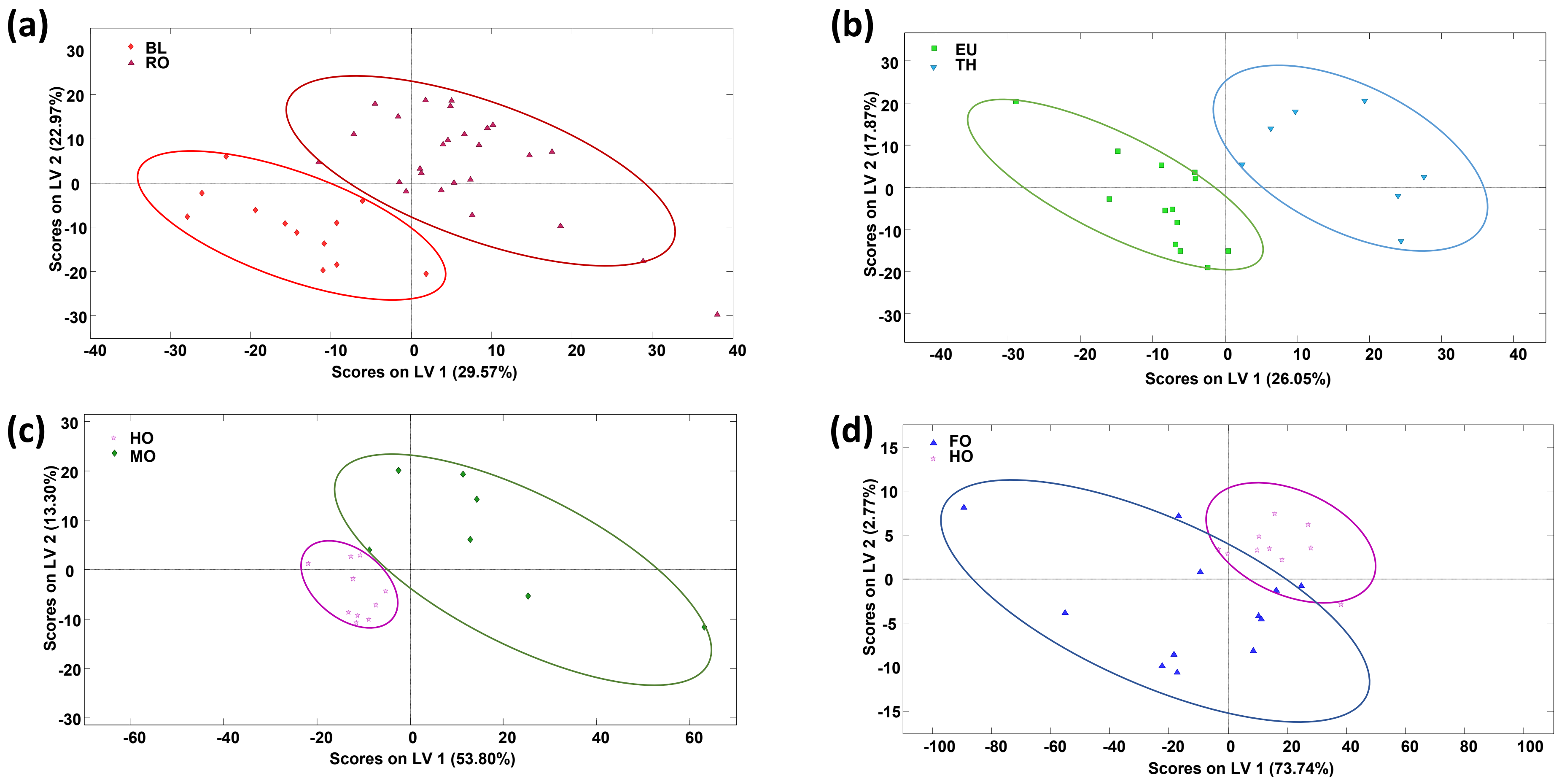

Figure S11. Supervised PLS-DA score plots of LV1 vs. LV2 when using off-line SPE HPLC-UV fingerprints (at 280 nm) as honey chemical descriptors of (a) orange/lemon vs rosemary blossom honeys (3 LVs were used to build the model), (b) thyme vs eucalyptus blossom honeys (2 LVs were used to build the model), (c) holm oak vs mountain honeydew honeys (2 LVs were used to build the model), and (d) holm oak vs forest honeydew honeys (2 LVs were used to build the model). BL: Orange/lemon blossom; EU: Eucalyptus; FO: Forest; HO: Holm oak; MO; Mountain; RO: Rosemary; TH: Thyme.
